# Common variants in genes coding for chemotherapy metabolizing enzymes, transporters, and targets: a case–control study of contralateral breast cancer risk in the WECARE Study

**DOI:** 10.1007/s10552-013-0237-6

**Published:** 2013-06-18

**Authors:** Jennifer D. Brooks, Sharon N. Teraoka, Leslie Bernstein, Lene Mellemkjær, Kathleen E. Malone, Charles F. Lynch, Robert W. Haile, Patrick Concannon, Anne S. Reiner, David J. Duggan, Katherine Schiermeyer, The WECARE Study Collaborative Group, Jonine L. Bernstein, Jane C. Figueiredo

**Affiliations:** 1 Department of Epidemiology and Biostatistics, Memorial Sloan-Kettering Cancer Center, 307 E 63rd Street, 3rd Floor, New York, NY USA; 2 Center for Public Health Genomics, University of Virginia, Charlottesville, VA USA; 3 Department of Biochemistry and Molecular Genetics, University of Virginia, Charlottesville, VA USA; 4 Department of Population Sciences, Beckman Research Institute of the City of Hope, Duarte, CA USA; 5 Research Department II, Institute of Cancer Epidemiology, Danish Cancer Society, Copenhagen, Denmark; 6 Program in Epidemiology, Division of Public Health Science, Fred Hutchinson Cancer Research Center, Seattle, WA USA; 7 Department of Epidemiology, University of Iowa College of Public Health, Iowa city, IA USA; 8 Department of Medicine, Division of Oncology, Stanford School of Medicine and the Stanford Cancer Institute, Stanford, CA USA; 9 Genetic Basis of Human Disease Division, Translational Genomic Research Institute, Phoenix, AZ USA

**Keywords:** Genetic variation, Chemotherapy, CMF, Contralateral breast cancer

## Abstract

**Purpose:**

Women who receive chemotherapy for a first primary breast cancer have been observed to have a reduced risk of contralateral breast cancer (CBC), however, whether the genetic profile of a patient modifies this protective effect is currently not understood. The purpose of this study is to investigate the impact of germline genetic variation in genes coding for drug metabolizing enzymes, transporters, and targets on the association between chemotherapy and risk of CBC.

**Methods:**

From the population-based Women’s Environment Cancer and Radiation Epidemiology (WECARE) Study, we included 636 Caucasian women with CBC (cases) and 1,224 women with unilateral breast cancer (controls). The association between common chemotherapeutic regimens, CMF and FAC/FEC, and risk of CBC stratified by genotype of 180 single nucleotide polymorphisms in 14 genes selected for their known involvement in metabolism, action, and transport of breast cancer chemotherapeutic agents, were determined using conditional logistic regression.

**Results:**

CMF (RR = 0.5, 95 % CI 0.4, 0.7) and FAC/FEC (RR = 0.7, 95 % CI 0.4, 1.0) are associated with lower CBC risk relative to no chemotherapy in multivariable-adjusted models. Here we show that genotype of selected genes involved in the metabolism and uptake of these therapeutic agents does not significantly alter the protective effect of either CMF or FAC/FEC on risk of CBC.

**Conclusion:**

The results of this study show that germline genetic variation in selected gene does not significantly alter the protective effect of CMF, FAC, and FEC on risk of CBC.

**Electronic supplementary material:**

The online version of this article (doi:10.1007/s10552-013-0237-6) contains supplementary material, which is available to authorized users.

## Background

Adjuvant chemotherapy is indicated in the clinical management of most premenopausal women and postmenopausal women with ER- tumors, improving disease-free and overall survival [1–5]. Studies have also shown that the risk of asynchronous contralateral breast cancer (CBC) is lower among individuals who receive chemotherapy for treatment of their first primary breast cancer [6, 7], with overall CBC risk reductions of 30–80 % reported in observational studies of women treated for breast cancer [7–12].

Germline genetic variation in drug metabolizing enzymes and transporters is thought to contribute to the observed inter-individual variation in treatment efficacy [13, 14]. The extent to which variation in these genes modifies the association between chemotherapy and risk of CBC is not known. Candidate genes can be classified into three main categories: phase I enzymes [e.g., cytochrome P450 (CYP) enzymes], phase II conjugation enzymes [e.g., glutathione S-transferases (GSTs)], and drug transporters (e.g., ABCB1). Together, these proteins influence the bio-activation, inactivation, and detoxification of a wide range of therapeutics [13]. The impact of variation in these genes on the association between chemotherapy and risk of CBC is not known.

In this study, we examined the impact of common single nucleotide variation in genes coding for drug metabolizing enzymes (*CYP1A1*, *CYP1B1*, *CYP2A6*, *CYP2B6*, *CYP2C9*, *CYP2D6*, *CYP3A4*, *CYP3A5*, *GSTM1*, *GSTM2*, *GSTP1*), targets (*DHFR*, *MTHFR*), and transporters (*ABCB1*), known to be involved in the metabolism and action of drugs commonly used in polychemotherapy regimens for breast cancer (e.g., cyclophosphamide, anthracyclines, and antimetabolites) [14, 15], on risk of CBC in the Women’s Environment Cancer and Radiation Epidemiology (WECARE) Study, a population-based case–control study of women with CBC (cases) and unilateral breast cancer (UBC) (controls).

## Methods

### Study population

Participants were identified through five population-based cancer registries: Los Angeles County Cancer Surveillance Program; Cancer Surveillance System of the Fred Hutchinson Cancer Research Center (Seattle); State Health Registry of Iowa; and the Cancer Surveillance Program of Orange County/San Diego-Imperial Organization for Cancer Control (Orange County/San Diego). These cancer registries contribute to the National Cancer Institute Surveillance, Epidemiology, and End Results (SEER) program. The fifth registry from which subjects were recruited was the Danish Breast Cancer Cooperative Group Registry, supplemented by data from the Danish Cancer Registry [16].

Details of CBC case and UBC control eligibility have been described previously [16]. Briefly, cases were women diagnosed prior to age 55 years, from 1985 to 2000, with invasive breast cancer that had not spread beyond regional lymph nodes. This had to be followed by a second in situ or invasive breast cancer diagnosed in the contralateral breast at least 1 year later. The ‘at-risk’ interval was defined as starting at the time of first breast cancer diagnosis and ending at reference date, that is, date of the second breast cancer diagnosis in cases (reference date) or the corresponding date in matched controls. Two controls were individually matched to each case on year of birth (in 5-year strata), year of diagnosis (in 4-year strata), registry region, and race/ethnicity. All women had to be alive at the time of contact and able to complete a telephone interview and donate a blood sample. Counter-matching based on registry-reported radiation treatment status was used to improve the statistical efficiency of the study design. Thus, for each radiation exposed case, one radiation exposed control and one unexposed control were selected from the relevant stratum; and for each unexposed case, two radiation exposed controls were selected [16].

Across the five cancer registries, 708 cases and 1,399 controls completed the study interview and provided a blood sample. Four individuals were excluded from the current analysis because they did not consent to genotyping beyond the initial *ATM*, *BRCA1*, and *BRCA2* mutation screening. To minimize the potential influence of ancestral differences in genotype frequencies, all analyses were restricted to Caucasian women (*n* = 1,933) as recorded by the cancer registry. Further exclusions were made after genotyping (see below).

### Data collection

The data collection protocol was approved by the institutional review board at each of the participating centers and by the Ethical Committee System in Denmark. Each woman provided written informed consent. Details of the study questionnaire have been published previously and included questions about known breast cancer risk factors [16]. Medical records, pathology reports, and hospital charts, in addition to self-reported data (collected during the telephone interview), were used to collect detailed treatment information (surgery, chemotherapy, hormonal therapy, radiation therapy) on the first primary breast cancer as well as during the at-risk period. Information collected on chemotherapy and hormonal therapy included dates of administration, reason for treatment (e.g., primary disease, recurrence), and type of drug. The most common chemotherapeutic regimens received by women in the WECARE Study population were cyclophosphamide (CTX), methotrexate (MTX), 5-fluorouracil (5FU) (CMF) (63 % of women treated with chemotherapy were treated with CMF) and 5FU, doxorubicin (Adriamycin^®^), CTX (FAC) or 5FU, epirubicin, CTX (FEC) (19 % of women treated with chemotherapy were treated with FAC/FEC) (Table 1). All other drug combinations were coded as ‘other’ chemotherapy. For the current analyses, a woman was classified as having received CMF or FAC/FEC if she received these combinations of drugs any time during her treatment for a first primary breast cancer and prior to the reference date.

**Table 1:** Characteristics of selected cases (women with asynchronous CBC) and controls (women with UBC only) from the WECARE Study population

Variable	Median (range)	Cases (CBC)	Controls (UBC)
		Median (range)	Median (range)
Age at first diagnosis (years)	46 (23–55)	46 (24–55)	46 (23–55)
Age at reference date (years)	51 (27–71)	51 (27–71)	51 (27–69)
Length of at-risk period (years)^a^	4 (1–16)	4 (1–16)	4 (1–16)

Variable	Level	Cases (CBC)		Controls (UBC)	
		*n*	%	*n*	%
Study site	Iowa	107	17	206	17
	Orange and San Diego Counties	105	17	202	17
	Los Angeles	154	24	290	24
	Seattle	94	15	187	15
	Denmark	176	28	339	28
Year of first diagnosis	1985–1988	221	35	422	35
	1989–1992	214	34	414	34
	1993–1996	160	25	309	25
	1997+	41	6	79	6
Chemotherapy	No	355	56	562	46
	Yes	281	44	662	54
CMF	Yes	155	24	439	36
FAC/FEC	Yes	61	10	119	10
Tamoxifen treatment	No	485	76	861	70
	Yes	139	22	338	28
	Unknown	12	2	25	2
Radiation treatment	Never	322	51	240	20
	Ever	314	49	984	80
Histology of first breast cancer	Lobular	82	13	120	10
	Other	554	87	1,104	90
Stage of first breast cancer	Localized	456	72	793	65
	Regional	180	28	431	35
ER Status of first breast cancer^b^	Positive	302	47	656	54
	Negative	165	26	288	24
	Other	169	27	280	23
PR Status of first breast cancer^b^	Positive	252	40	536	44
	Negative	144	23	270	22
	Other	240	38	418	34
Menopausal status/age at menopause at first diagnosis	Premenopausal	468	74	919	75
	Postmenopausal age <45	84	13	183	15
	Postmenopausal age ≥45	83	13	118	10
	Unknown	1	0.2	4	0
Family history of breast cancer	None	420	66	954	78
	≥1 First-degree relative	205	32	246	20
	Adopted	11	2	24	2

Includes Caucasian women with SNP call rates ≥95 %, without significant African or Asian ancestry with complete information on tamoxifen treatment and genotype data from both the Omni1-Quad and custom BeadChip platforms (636 CBC cases and 1,224 UBC controls)

CBC = asynchronous contralateral breast cancer; UBC = unilateral breast cancer; CMF = cyclophosphamide, methotrexate, 5-fluorouracil; FAC/FEC = cyclophosphamide, doxorubicin/epirubicin, 5-fluorouracil chemotherapy; ER = estrogen receptor, PR = progesterone receptor

^a^Beginning 1 year after first diagnosis extending to the reference date (date of second diagnosis in cases)

^b^Refers to receptor status of the first primary breast cancer. The ‘other’ category consists of women for whom no lab test was given, the test was given and the results are unknown or the test was given and the results were borderline

### Genotyping

Genes were selected for their known involvement in the metabolism, action, and transport of chemotherapeutic agents commonly used to treat breast cancer. A list of genes and their associated drugs can be found in Table 2.

**Table 2:** Candidate genes coding for selected drug metabolizing enzymes, targets, and transporters

Drugs	Genes of interest
Cyclophosphamide	*CYP1A1*, *CYP1B1*, *CYP2A6*, *CYP2B6*, *CYP2C9*, *CYP2D6*, *CYP3A4*, *CYP3A5*, *GSTM1*, *GSTM2*, *GSTP1*
Methotrexate, 5-fluorouracil	*DHFR*, *MTHFR*, *ABCB1*
Doxorubicin (adriamycin), epirubicin	*GSTM1*, *GSTM2*, *GSTP1*, *ABCB1*

DNA was prepared from blood samples by red cell lysis and standard methods of phenol/chloroform extraction. Samples were genotyped with Illumina’s HumanOmni1-Quad BeadChip (Illumina Inc., San Diego, CA, USA) as part of the WECARE Study’s GWAS effort. Default Omni1-Quad cluster definitions supplied by Illumina were used to call genotypes, and single nucleotide polymorphisms (SNPs) with GenTrain scores <0.36 were considered ‘no calls,’ and samples with call rates <95 % were excluded in addition to other exclusion criteria described below. Each 96 well plate included one inter-plate positive quality control sample (NA06990—Coriell Cell Repositories). In addition, 38 blinded and 46 unblinded quality controls replicates from the study sample were genotyped. Concordance rates for both the Coriell and study sample replicates were high: >99.99 %.

Additional genotyping in these genes was performed to broaden gene coverage. SNP lists from the HapMap project (http://hapmap.ncbi.nlm.nih.gov/) were imported into Tagger (in Haploview) [17], and haplotype tagging SNPs (tagSNPs) were selected based on patterns of linkage disequilibrium (LD) with boundaries suggested by Gabriel et al. [18]. tagSNPs were selected based on pairwise tagging with a minimum *r* ^2^ of 0.90. Multiplex SNP genotyping was carried out using the Illumina Golden Gate™ assay on custom BeadChips (Illumina Inc., San Diego, CA, USA). Laboratory methods and sample control measures have been described previously [19].

The CYP2D6*4 (rs3892097) variant was genotyped by a modified MGB Eclipse probe assay (Epoch Biosciences, ELITech Group, Paris, France). The outer primers designed to exclude pseudogenes were 5′ AGCCTGCCCCAGCCAAGGGAGC 3′ and 5′ CTCGGTCTCTCGCTCCGCAC 3′. The internal primers were designed by Epoch Biosciences to encompass the SNP: 5′ AATAAATCATAACCCCTTACCCGCATCTC 3′ and 5′ GATCACGTTGCTCACGGCTTTGTCCAAGAG 3′. DNA was amplified using the standard Eclipse protocol except that in the first 15 of 50 cycles, and the annealing temperature was increased by ten degrees to 68 °C. This method resulted in 100 % concordance of genotypes among the 24 % blinded, re-sampled DNAs. A subset of samples (17 %) was confirmed by a second method, allele-specific tetra-primer PCR, and separation of the allele-specific fragment sizes on 1 % agarose [20].

Quality control steps applied to the genome-wide association study (GWAS) data lead to further subject exclusions: (a) Women with SNP call rates <95 % were excluded (*n* = 22); (b) Population stratification was investigated using EIGENSTRAT [21]; using the first two principal components, 9 outliers with significant African or Chinese ancestry were identified for exclusion; and (c) 14 additional participants were excluded due to incomplete matched sets. Identity by descent was examined using PLINK [22] identifying 3 pairs of sisters, including one pair of identical twins. These women were not excluded from the analysis. An additional 28 subjects were excluded because they had >5 % missing genotypes on the SNP BeadChips. Analyses are based on the remaining 1,860 participants (636 CBC cases and 1,224 UBC controls) with genotype data from both the Omni1-Quad and custom bead chips platforms.

Within the selected genes of interest, 260 SNPs were genotyped on the OMNI platform, 27 SNPs on the SNP BeadChip, and rs3892097 (CYP2D6*4) on a modified MGB Eclipse probe assay (for a total of 287 genotyped SNPs). SNPs with >10 % missing (*n* = 16) and those that were monomorphic (*n* = 87) were excluded. Although Hardy–Weinberg equilibrium may not strictly apply since all participants in the study were affected with breast cancer, 4 SNPs deviating from Hardy–Weinberg equilibrium (*p* < 0.001) were also excluded. This left 180 SNPs in or near 14 genes to be included in the analyses: 1 SNP in *CYP1A1*, 4 in *CYP1B1*, 2 in *CYP2A6*, 6 in *CYP2B6*, 4 in *CYP2C9*, 9 in *CYP2D6*, 26 in *CYP3A4*, 33 in *CYP3A5*, 13 in *DHFR*, 3 in *GSTM1*, 1 in *GSTM2*, 22 in *GSTP1*, 54 in *MTHFR*, and 2 in *ABCB1* (Online Resource 1).

### Statistical analysis

In analyses examining the impact of genotype on the association between CMF and risk of CBC, the chemotherapy regimen variable was coded as CMF, other chemotherapy regimens, and no chemotherapy. Similar coding was used for FAC/FEC analyses. In all instances, the comparison group was women who did not receive chemotherapy. In the CMF analyses, FAC/FEC was coded as ‘other chemotherapy’ and vice versa. SNPs in genes that code for enzymes involved in the metabolism or action of cyclophosphamide, methotrexate, 5-fluorouracil, or doxorubicin/epirubicin were included in the analyses (Table 2). Based on the combination of drugs used in either regimen, the same SNPs were included in the CMF and FAC/FEC analyses.

Rate ratios (RR) and 95 % confidence intervals (CI) were estimated using conditional logistic regression to examine the association between chemotherapeutic regimen (CMF or FAC/FEC) and risk of CBC, stratified by genotype for each SNP using the dominant model [0 (homozygous wild-type), 1 (heterozygous and homozygous variant)]. Models were run adjusting for age at first breast cancer diagnosis and included an ‘offset term’ (i.e., log weight ‘covariate’ in the model where the coefficient of this log weight is fixed at one [16]), taking into account the sampling probabilities of the counter-matching. Multi-variable adjusted models were also run including adjustment for age at first diagnosis, family history, stage and histology of first primary breast cancer, and other treatments (hormonal therapy and radiation therapy). The likelihood ratio test was used to test for heterogeneity of treatment effect across genotypes.

Age and multivariable-adjusted [as described above] analyses were also conducted to confirm the association between chemotherapy and CBC risk in the subgroup of women included in the current analyses [1,860 (88 %) of the 2,107 total number of women in the WECARE Study].

A conservative Bonferroni correction was used to determine the multiple comparison cut-point (*α* = 0.0003, obtained from (0.05/180 SNPs) at which results were considered statistically significant. All analyses were conducted using SAS 9.2 (SAS Institute Inc., Cary, NC, USA). Figures were generated using Microsoft^®^ Excel 2007.

## Results

**Fig. 1:**
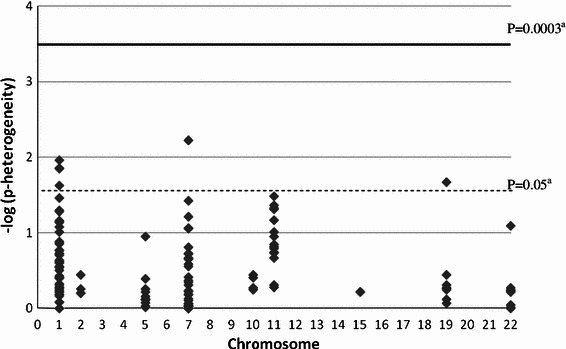
Log_10_ *p* value for heterogeneity (multivariable-adjusted models) of the association between CMF treatment regimen and risk of CBC for 180 SNPs. ^a^The *dashed line* shows the *p* value cut-off of 0.05 and the *solid line* the Bonferroni-corrected *p* value cut-off of 0.0003

**Fig. 2:**
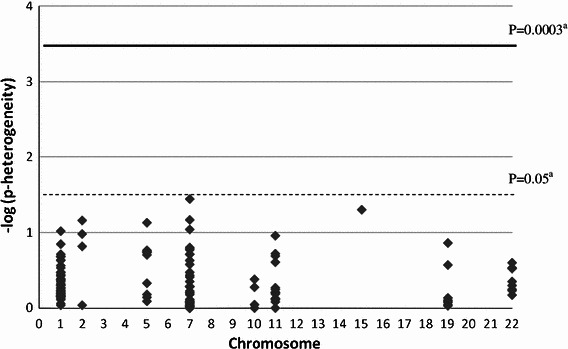
Log_10_ *p* value for heterogeneity (multivariable-adjusted models) of the association between FAC/FEC treatment regimen and risk of CBC for 180 SNPs. ^a^The *dashed line* shows the *p* value cut-off of 0.05 and the *solid line* the Bonferroni-corrected *p* value cut-off of 0.0003

Selected characteristics of the eligible WECARE Study population are shown in Table 1. Cases and controls were similar for all matching characteristics. In multivariable-adjusted models, both CMF (RR = 0.5, 95 % CI 0.4, 0.7) and FAC/FEC (RR = 0.7, 95 % CI 0.4, 1.0) are associated with lower risk of CBC relative to no chemotherapy. In stratified analyses using the dominant model, chemotherapy was protective with respect to risk of CBC, regardless of genotype. Figures 1 and 2 show the −log_10_(*p* for heterogeneity) for each SNP, grouped by chromosome, and for CMF and FAC/FEC analyses, respectively. Results showing the association between CMF and FAC/FEC treatments and risk of CBC stratified by genotype for all SNPs did not differ in age and multivariable adjusted models and can be found in Online Resource 2 and Online Resource 3, respectively. Findings from some commonly studied candidate SNPs are reported below.

In multivariable-adjusted models, the variants rs1801133 and rs1801131 in *MTHFR*, known to be associated with altered enzyme activity, did not significantly modify the reduction in CBC risk seen with CMF (RR = 0.5, 95 % CI 0.3, 0.8 in rs1801133 wild-type compared to RR = 0.7, 95 % CI 0.4, 1.1 in women who are heterozygous or homozygous for the rs1801133 variant, *p* for heterogeneity =0.29, and RR = 0.8, 95 % CI 0.5, 1.3 in rs1801131 wild-type compared to RR = 0.4, 95 % CI 0.3, 0.7 in women who are heterozygous or homozygous for the rs1801131 variant, *p* for heterogeneity =0.01).

Rs1695 in *GSTP1* also did not significantly modify the effect of chemotherapy on risk of CBC (RR = 0.5, 95 % CI 0.3, 0.9 in rs1695 wild-type compared to RR = 0.7, 95 % CI 0.4, 1.0 in women who are heterozygous or homozygous for the rs1695 variant, *p* for heterogeneity =0.49 in women receiving CMF, and RR = 0.9, 95 % CI 0.5, 1.8 in rs1695 wild-type compared to RR = 0.6, 95 % CI 0.3, 1.1 in women who are heterozygous or homozygous for the rs1695 variant, *p* for heterogeneity =0.25 in women receiving FAC/FEC).

Similarly, the association between chemotherapy and risk of CBC did not differ when stratified by CYP3A4*1B (rs2740574) genotype (RR = 0.6, 95 % CI 0.4, 0.9 in rs2740574 wild-type compared to RR = 1.0, 95 % CI 0.4, 2.7 in women who are heterozygous or homozygous for the rs2740574 variant, *p* for heterogeneity =0.22 in women receiving CMF, and RR = 0.7, 95 % CI 0.4, 1.2 in rs2740574 wild-type compared to RR = 0.7, 95 % CI 0.2, 2.6 in women who are heterozygous or homozygous for the rs2740574 variant, *p* for heterogeneity =0.91 in women receiving FAC/FEC).

## Discussion

Chemotherapy reduces the risk of CBC [6–12]; however, the impact of germline genetic variation in drug metabolizing enzymes, targets, and transporters on this association has not been investigated. Consistent with our prior publication [8], chemotherapy was associated with a lower risk of CBC. Here, we show that variation in these selected genes did not alter this protective effect of chemotherapy on risk of CBC in a large, well-characterized study population. This is the first study to specifically address the association between genetic variants, chemotherapy, and risk of CBC and to show that chemotherapy is protective with respect to CBC risk, despite differences in the genetic profiles of the genes investigated here.

CTX, a widely used nitrogen mustard alkylating agent, is a component of both the CMF and FAC/FEC regimens and the most common agent received by the WECARE Study population. The pharmacokinetics of CTX are highly variable (reviewed in [23]). CTX is administered as an inactive pro-drug that requires metabolization by several cytochrome P450 enzymes including CYP2B6 [24], CYP2C9 [25], and CYP3A4 [26] with minor contributions from CYP2A6, CYP2C8, and CYP2C19 (reviewed in [27]), to its active metabolite 4-hydroxycyclophosphamide (4OHCTX), which is further detoxified by the phase II enzymes GSTs [28]. Patient characteristics, including weight and age, influence treatment efficacy, but some variation in clinical response may also be attributed to germline genetic variation in these phase I and phase II enzymes. Comparison between studies is complicated by the inclusion of different SNPs in different genes. Prior studies have shown that some variants in CYPs and GSTs can alter the pharmacokinetics of CTX metabolism [27, 29, 30] and influence clinical response and toxicity of CTX-based chemotherapies [31–38]. Other studies have found no association between genetic variants in these genes and CTX pharmacokinetics [39] or outcome [40]. Our study of some of these same variants found that genotype did not alter the association between CTX-based chemotherapies and risk of CBC.

MTX and 5FU are antimetabolites that interfere with cellular metabolism. MTX acts by inhibiting two enzymes: dihydrofolate reductase (DHFR) and thymidylate synthase (TS). 5FU, as an anti-folate metabolite, has several cytotoxic mechanisms [41]. MTHFR is a central regulatory enzyme in folate metabolism and has known variants that impact enzyme function (e.g., rs1801131, rs1801133). These variants have been shown to alter methotrexate toxicity (reviewed in [42]). Variation in *MTHFR* has also been shown to increase sensitivity to 5FU and decrease sensitivity to MTX in breast cancer cell lines [43], increase risk of mortality after chemotherapy for breast cancer [44], and reduce 5FU response in colorectal cancer [45–47]. Paré et al. [48] found no association between variation in *MTHFR* and disease-free survival in breast cancer patients who received CMF or FEC. Our study also found that variants in *MTHFR*, including those known to influence enzyme function, did not modify the effect of chemotherapy on risk of CBC.

Anthracyclines [e.g., doxorubicin, epirubicin (the 4′-epimer of doxorubicin)] have multiple anti-cancer mechanisms including DNA intercalation, generation of free-radicals, and disruption of topoisomerase II-mediated DNA repair [49]. Doxorubicin is metabolized in the liver by the phase I enzymes aldoketoreductases and carbonyl reductases to an active metabolite, doxorubicinol which is then detoxified by phase II GSTs. Gor et al. [36] examined the impact of variation in *CYP3A4*, *CYP3A5*, *CYP2B6*, *CYP2D6*, CYP2C9, *GSTP1*, *GSTM1*, and *GSTT1* and found that women carrying at least one CYP3A4*1B variant allele (rs2740574) had significantly shorter disease-free survival than wild-type women. The same variants were examined by Yao et al. [40], and they were not able to reproduce this association. Another study found that variants in *CYP2B6* (rs192709 and rs3211371) were associated with an increased risk of dose delay in women receiving AC (doxorubicin, cyclophosphamide) chemotherapy. Other SNPs in this same gene (rs8192709, rs3745274, rs2279343) were associated with worse outcome [37]. Our study found that variation in *CYP2B6* did not modify the effect of FAC/FEC on risk of CBC.

Doxorubicin and MTX are also substrates of P-glycoprotein, an efflux transporter that is the product of the *ABCB1* [multi-drug resistance (*MDR*-*1*)] gene (reviewed in [50, 51]). Lal et al. [52] found that SNPs in *ABCB1* increased drug exposure by decreasing its clearance. Variation in *ABCB1* has also been associated with clinical response and overall survival in women receiving doxorubicin-based chemotherapy [53–55]. Studies examining the impact of variation in *GSTP1* have been mixed [56, 57].

The strengths of this study include the population-based design, the large number of women with CBC, enabling the examination of CBC as an outcome, and the extensive review of patient medical records and questionnaire data, to obtain detailed treatment information. A limitation of the tagSNP approach used here is that it does not address the impact of less common or rare variants (MAF < 5 %), SNPs not in LD with typed variants, insertions/deletions, epigenetic modifications, and copy number variations, on treatment response. Further, complete gene coverage was not achieved for all genes, and in some cases, a candidate SNP approach was used (e.g., *CYP1A1*). It is possible that un-typed variants in these candidate genes and variation in genes not included in the current analysis could modify the effect of treatment on risk of CBC. A further limitation of this study is that for variants with a low minor allele frequency or modest effects on the association between chemotherapy and risk of CBC, our power is reduced.

## Conclusion

This is the first study to specifically address the impact of germline genetic variation on the association between chemotherapy and risk of CBC. The results of this study suggest that chemotherapy (CMF and FAC/FEC) is associated with a lower risk of CBC regardless of genetic variation in selected genes that code for proteins involved in the metabolism of these commonly used chemotherapeutic agents.

### Electronic supplementary material

Below is the link to the electronic supplementary material.

Supplementary material 1 (XLS 51 kb)

Supplementary material 2 (XLS 166 kb)

Supplementary material 3 (XLS 166 kb)

## Appendix: The WECARE Study Collaborative Group

Memorial Sloan-Kettering Cancer Center (New York, NY): Jonine L. Bernstein Ph.D. (WECARE Study P.I.), Colin Begg. Ph.D., Jennifer D. Brooks Ph.D., Marinela Capanu Ph.D., Xiaolin Liang M.D., Anne S. Reiner M.P.H., Irene Orlow Ph.D, Robert Klein Ph.D. (Co-investigator), Ken Offit M.D. (Co-investigator); Meghan Woods M.P.H.;

Beckman Research Institute, City of Hope National Medical Center (Duarte, CA): Leslie Bernstein Ph.D. (sub-contract P.I.);

Cancer Prevention Institute of California (Fremont, CA): Esther M. John Ph.D. (Sub-contract PI);

Danish Cancer Society (Copenhagen, Denmark): Jørgen H. Olsen M.D. DMSc. (Sub-contract P.I.), Lene Mellemkjær Ph.D.;

Fred Hutchinson Cancer Research Center (Seattle, WA): Kathleen E. Malone Ph.D. (Sub-contract P.I.);

National Cancer Institute (Bethesda, MD): Daniela Seminara Ph.D. M.P.H;

National Council on Radiation Protection and Measurements (Bethesda, MD) and Vanderbilt University (Nashville, TN): John D. Boice Jr. Sc.D. (Sub-contract P.I.);

New York University (New York, NY): Roy E. Shore Ph.D., Dr. P.H. (Sub-contract P.I.);

Samuel Lunenfeld Research Institute, Mount Sinai Hospital (Toronto, Canada): Julia Knight, Ph.D. (Sub-contract P.I.), Anna Chiarelli Ph.D. (Co-Investigator);

Stanford School of Medicine (Stanford, CA): Robert W. Haile Dr. P.H. (Sub-contract P.I.), Anh T. Diep (Co-Investigator), Nianmin Zhou, M.D.;

Translational Genomics Research Institute (TGen) (Phoenix, AZ): David Duggan Ph.D. (Sub-contract P.I.);

University of Florida (Gainesville, FA): Patrick Concannon, Ph.D. (Sub-contract P.I.), Sharon Teraoka, Ph.D. (Co-Investigator);

University of Iowa (Iowa City, IA): Charles F. Lynch M.D., Ph.D. (Sub-contract P.I.), Michele West, Ph.D.;

University of Southern California (Los Angeles, CA): Daniel Stram Ph.D. (Sub-contract P.I.), Duncan C. Thomas Ph.D. (Co-Investigator), Dave Conti Ph.D., Shanyan Xue M.D., Evgenia Ter-Karapetova;

University of Texas, M.D. Anderson Cancer Center (Houston, TX): Marilyn Stovall Ph.D. (Sub-contract P.I.), Susan Smith M.P.H. (Co-Investigator).
